# Supplementary material for: Migration Modeling as a Valuable Tool for Exposure Assessment and Risk Characterization of Polyethylene Terephthalate Oligomers
Source: Molecules. 2022 Dec 25;28(1):173. doi: 10.3390/molecules28010173 (PMC9822255; doi:10.3390/molecules28010173)
Supplement: Supplementary file 1 [file molecules-28-00173-s001.zip › molecules-2087139-supplementary.pdf]

Supplementary Material:

Migration modeling as a valuable tool for exposure assessment and risk characterization of polyethylene terephthalate oligomers

Verena N. Schreier[1,2], Alex Odermatt[1,2], Frank Welle[3,*]

[1] Division of Molecular and Systems Toxicology, Department of Pharmaceutical Sciences, University of Basel, Basel, Switzerland
[2] Swiss Centre for Applied Human Toxicology (SCAHT), University of Basel, Basel, Switzerland
[3] Product Safety and Analytics, Fraunhofer Institute for Process Engineering and Packaging (IVV), Freising, Germany

*Correspondence: Frank Welle (frank.welle@ivv.fraunhofer.de)

Table of Contents

Table S1 2
Table S2 3
Figure S1 5
Figure S2 6
Figure S3 7
Figure S4 8
Figure S5 9
Figure S6 10
Figure S7 11
Figure S8 12
Figure S9 13
References.. 14

Table S1. SMILES and CAS Registry Numbers (CAS RN) of PET oligomers used for migration predictions. Abbreviations: C (cyclic), L (linear), TPA (terephthalic acid), EG (ethylene glycol), DEG (diethylene glycol).

PET oligomer (CAS RN)	SMILES
C[TPA+EG] (7337-79-3)	O=C1OCCOC(=O)C2=CC=C1C=C2
C[TPA+EG]2 (24388-68-9)	O=C1OCCOC(=O)C2=CC=C(C=C2)C(=O)OCCOC(=O)C3=CC=C1C=C3
C[TPA+EG]3 (7441-32-9)	O=C1OCCOC(=O)C2=CC=C(C=C2)C(=O)OCCOC(=O)C3=CC=C(C=C3)C(=O)OCCOC(=O)C4=CC=C1C=C4
C[TPA+EG]4 (16104-96-4)	O=C1OCCOC(=O)C2=CC=C(C=C2)C(=O)OCCOC(=O)C3=CC=C(C=C3)C(=O)OCCOC(=O)C4=CC=C(C=C4)C(=O)OCCOC(=O)C5=CC=C1C=C5
C[TPA+EG]5 (16104-97-5)	O=C1OCCOC(=O)C2=CC=C(C=C2)C(=O)OCCOC(=O)C3=CC=C(C=C3)C(=O)OCCOC(=O)C4=CC=C(C=C4)C(=O)OCCOC(=O)C5=CC=C(C=C5)C(=O)OCCOC(=O)C6=CC=C1C=C6
C[TPA+EG]6 (29644-29-9)	O=C1OCCOC(=O)C2=CC=C(C=C2)C(=O)OCCOC(=O)C3=CC=C(C=C3)C(=O)OCCOC(=O)C4=CC=C(C=C4)C(=O)OCCOC(=O)C5=CC=C(C=C5)C(=O)OCCOC(=O)C6=CC=C(C=C6)C(=O)OCCOC(=O)C7=CC=C1C=C7
C[TPA+EG]7 (29668-12-0)	O=C1OCCOC(=O)C2=CC=C(C=C2)C(=O)OCCOC(=O)C3=CC=C(C=C3)C(=O)OCCOC(=O)C4=CC=C(C=C4)C(=O)OCCOC(=O)C5=CC=C(C=C5)C(=O)OCCOC(=O)C6=CC=C(C=C6)C(=O)OCCOC(=O)C7=CC=C(C=C7)C(=O)OCCOC(=O)C8=CC=C1C=C8
C[TPA+EG]8 (42245-76-1)	O=C1OCCOC(=O)C2=CC=C(C=C2)C(=O)OCCOC(=O)C3=CC=C(C=C3)C(=O)OCCOC(=O)C4=CC=C(C=C4)C(=O)OCCOC(=O)C5=CC=C(C=C5)C(=O)OCCOC(=O)C6=CC=C(C=C6)C(=O)OCCOC(=O)C7=CC=C(C=C7)C(=O)OCCOC(=O)C8=CC=C(C=C8)C(=O)OCCOC(=O)C9=CC=C1C=C9
C[TPA+DEG] (18189-01-0)	O=C1OCCOCCOC(=O)C2=CC=C1C=C2
C[TPA+EG]+[TPA+DEG] (29278-57-7)	O=C1OCCOC(=O)C2=CC=C(C=C2)C(=O)OCCOCCOC(=O)C3=CC=C1C=C3
C[TPA+EG]2+[TPA+DEG] (873422-64-1)	O=C1OCCOC(=O)C2=CC=C(C=C2)C(=O)OCCOCCOC(=O)C3=CC=C(C=C3)C(=O)OCCOC(=O)C4=CC=C1C=C4
C[TPA+EG]3+[TPA+DEG] (2222729-29-3)	O=C(C1=CC=C(C(OCCOCCOC(C(C=C2)=CC=C2C(OCCOC(C3=CC=C4C=C3)=O)=O)=O)=O)C=C1)OCCOC(C(C=C5)=CC=C5C(OCCOC4=O)=O)=O
C[TPA+EG]4+[TPA+DEG] (not available)	O=C(C1=CC=C(C(OCCOCCOC(C(C=C2)=CC=C2C(OCCOC(C3=CC=C4C=C3)=O)=O)=O)=O)C=C1)OCCOC(C(C=C5)=CC=C5C(OCCOC(C(C=C6)=CC=C6C(OCCOC4=O)=O)=O)=O)=O
C[TPA+EG]5+[TPA+DEG] (not available)	O=C(OCCOC(C1=CC=C(C=C1)C(OCCOC(C2=CC=C(C=C2)C(OCCOC(C3=CC=C(C=C3)C(OCCOC(C4=CC=C(C=C4)C(OCCO5)=O)=O)=O)=O)=O)=O)=O)=O)C(C=C6)=CC=C6C(OCCOCCOC(C(C=C7)=CC=C7C5=O)=O)=O
C[TPA+DEG]2 (16104-98-6)	O=C1OCCOCCOC(=O)C2=CC=C(C=C2)C(=O)OCCOCCOC(=O)C3=CC=C1C=C3
C[TPA+EG]+[TPA+DEG]2 (not available)	O=C(C1=CC=C(C2=O)C=C1)OCCOC(C3=CC=C(C(OCCOCCOC(C4=CC=C(C(OCCOCCO2)=O)C=C4)=O)=O)C=C3)=O
C[TPA+EG]2+[TPA+DEG]2 (not available)	O=C(C1=CC=C(C(OCCOC(C2=CC=C(C(OCCOCCOC(C3=CC=C4C=C3)=O)=O)C=C2)=O)=O)C=C1)OCCOC(C5=CC=C(C(OCCOCCOC4=O)=O)C=C5)=O
C[TPA+EG]3+[TPA+DEG]2 (not available)	O=C(OCCOCCOC(C1=CC=C(C(OCCOCCOC(C2=CC=C(C3=O)C=C2)=O)=O)C=C1)=O)C4=CC=C(C(OCCOC(C5=CC=C(C(OCCOC(C6=CC=C(C(OCCO3)=O)C=C6)=O)=O)C=C5)=O)=O)C=C4
C[TPA+EG]4+[TPA+DEG]2 (not available)	O=C(C1=CC=C(C=C1)C(OCCOC(C2=CC=C(C=C2)C(OCCOC(C3=CC=C4C=C3)=O)=O)=O)=O)OCCOCCOC(C5=CC=C(C=C5)C(OCCOCCOC(C6=CC=C(C=C6)C(OCCOC(C(C=C7)=CC=C7C(OCCOC4=O)=O)=O)=O)=O)=O)=O
C[TPA+EG]+[TPA+DEG]3 (not available)	O=C(C1=CC=C(C(OCCOCCOC(C2=CC=C(C(OCCOC(C3=CC=C4C=C3)=O)=O)C=C2)=O)=O)C=C1)OCCOCCOC(C(C=C5)=CC=C5C(OCCOCCOC4=O)=O)=O
L[TPA+EG] (1137-99-1)	O=C(O)C1=CC=C(C=C1)C(=O)OCCO
L[TPA+EG]2 (23186-89-2)	O=C(O)C1=CC=C(C=C1)C(=O)OCCOC(=O)C2=CC=C(C=C2)C(=O)OCCO
L[TPA+EG]3 (16958-96-6)	O=C(O)C1=CC=C(C=C1)C(=O)OCCOC(=O)C2=CC=C(C=C2)C(=O)OCCOC(=O)C3=CC=C(C=C3)C(=O)OCCO
L[TPA+EG]4 (1497479-05-6)	O=C(O)C1=CC=C(C=C1)C(=O)OCCOC(=O)C2=CC=C(C=C2)C(=O)OCCOC(=O)C3=CC=C(C=C3)C(=O)OCCOC(=O)C4=CC=C(C=C4)C(=O)OCCO
L[TPA+EG]5 (51799-34-9)	O=C(O)C1=CC=C(C=C1)C(=O)OCCOC(=O)C2=CC=C(C=C2)C(=O)OCCOC(=O)C3=CC=C(C=C3)C(=O)OCCOC(=O)C4=CC=C(C=C4)C(=O)OCCOC(=O)C5=CC=C(C=C5)C(=O)OCCO
L[TPA+EG]6 (not available)	O=C(C1=CC=C(C(OCCOC(C2=CC=C(C(OCCOC(C3=CC=C(C(OCCOC(C4=CC=C(C(OCCOC(C5=CC=C(C(OCCOC(C6=CC=C(C(OCCO)=O)C=C6)=O)=O)C=C5)=O)=O)C=C4)=O)=O)C=C3)=O)=O)C=C2)=O)=O)C=C1)O
L[TPA+EG]7 (not available)	O=C(C1=CC=C(C(OCCOC(C2=CC=C(C(OCCOC(C3=CC=C(C(OCCOC(C4=CC=C(C(OCCOC(C5=CC=C(C(OCCOC(C6=CC=C(C(OCCOC(C7=CC=C(C(OCCO)=O)C=C7)=O)=O)C=C6)=O)=O)C=C5)=O)=O)C=C4)=O)=O)C=C3)=O)=O)C=C2)=O)=O)C=C1)O
L[TPA+EG]8 (not available)	O=C(C1=CC=C(C(OCCOC(C2=CC=C(C(OCCOC(C3=CC=C(C(OCCOC(C4=CC=C(C(OCCOC(C5=CC=C(C(OCCOC(C6=CC=C(C(OCCOC(C7=CC=C(C(OCCOC(C8=CC=C(C(OCCO)=O)C=C8)=O)=O)C=C7)=O)=O)C=C6)=O)=O)C=C5)=O)=O)C=C4)=O)=O)C=C3)=O)=O)C=C2)=O)=O)C=C1)O
L[TPA+DEG] (65087-23-2)	O=C(O)C1=CC=C(C=C1)C(=O)OCCOCCO

L[TPA+DEG]+EG (65133-69-9)	OCCOC(C1=CC=C(C(OCCOCCO)=O)C=C1)=O
L[TPA+EG]+[TPA+DEG] (not available)	O=C(C1=CC=C(C(OCCOC(C2=CC=C(C(OCCOCCO)=O)C=C2)=O)=O)C=C1)O
L[TPA+EG]2+[TPA+DEG] (not available)	O=C(C1=CC=C(C(OCCOC(C2=CC=C(C(O)=O)C=C2)=O)=O)C=C1)OCCOC(C3=CC=C(C(OCCOCCO)=O)C=C3)=O
L[TPA+EG]3+[TPA+DEG] (not available)	O=C(OCCOC(C1=CC=C(C=C1)C(OCCOC(C2=CC=C(C=C2)C(OCCOCCO)=O)=O)=O)=O)C3=CC=C(C=C3)C(OCCOC(C4=CC=C(C=C4)C(O)=O)=O)=O
L[TPA+EG]4+[TPA+DEG] (not available)	O=C(OCCOC(C1=CC=C(C(OCCOC(C2=CC=C(C(O)=O)C=C2)=O)=O)C=C1)=O)C3=CC=C(C(OCCOC(C4=CC=C(C(OCCOC(C5=CC=C(C(OCCOCCO[H])=O)C=C5)=O)=O)C=C4)=O)=O)C=C3
L[TPA+EG]5+[TPA+DEG] (not available)	O=C(OCCOC(C1=CC=C(C(OCCOCCOC(C2=CC=C(C(O)=O)C=C2)=O)=O)C=C1)=O)C3=CC=C(C(OCCOC(C4=CC=C(C(OCCOC(C5=CC=C(C(OCCOC(C6=CC=C(C(OCCO[H])=O)C=C6)=O)=O)C=C5)=O)=O)C=C4)=O)=O)C=C3
L[TPA+DEG]2 (not available)	[H]OCCOCCOC(C1=CC=C(C(OCCOCCOC(C2=CC=C(C(O)=O)C=C2)=O)=O)C=C1)=O
L[TPA+EG]+[TPA+DEG]2 (not available)	O=C(C1=CC=C(C(OCCOC(C2=CC=C(C(O)=O)C=C2)=O)=O)C=C1)OCCOCCOC(C3=CC=C(C(OCCOCCO)=O)C=C3)=O
L[TPA+EG]2+[TPA+DEG]2 (not available)	O=C(C1=CC=C(C(OCCOC(C2=CC=C(C(O)=O)C=C2)=O)=O)C=C1)OCCOC(C3=CC=C(C(OCCOCCOC(C4=CC=C(C(OCCOCCO)=O)C=C4)=O)=O)C=C3)=O
L[TPA+EG]3+[TPA+DEG]2 (not available)	O=C(OCCOC(C1=CC=C(C(OCCOC(C2=CC=C(C(O)=O)C=C2)=O)=O)C=C1)=O)C3=CC=C(C(OCCOC(C4=CC=C(C(OCCOCCOC(C5=CC=C(C(OCCOCCO[H])=O)C=C5)=O)=O)C=C4)=O)=O)C=C3
L[TPA+EG]4+[TPA+DEG]2 (not available)	O=C(C1=CC=C(C=C1)C(OCCOC(C2=CC=C(C=C2)C(OCCOC(C3=CC=C(C=C3)C(OCCO)=O)=O)=O)=O)=O)OCCOCCOC(C4=CC=C(C=C4)C(OCCOCCOC(C5=CC=C(C=C5)C(OCCOC(C(C=C6)=CC=C6C(O)=O)=O)=O)=O)=O)=O
L[TPA+EG]+EG (959-26-2)	O=C(OCCO)C1=CC=C(C=C1)C(=O)OCCO
L[TPA+EG]2+EG (2144-69-6)	O=C(OCCO)C1=CC=C(C=C1)C(=O)OCCOC(=O)C2=CC=C(C=C2)C(=O)OCCO
L[TPA+EG]3+EG (16033-73-1)	O=C(OCCO)C1=CC=C(C=C1)C(=O)OCCOC(=O)C2=CC=C(C=C2)C(=O)OCCOC(=O)C3=CC=C(C=C3)C(=O)OCCO
L[TPA+EG]4+EG (34298-51-6)	O=C(OCCO)C1=CC=C(C=C1)C(=O)OCCOC(=O)C2=CC=C(C=C2)C(=O)OCCOC(=O)C3=CC=C(C=C3)C(=O)OCCOC(=O)C4=CC=C(C=C4)C(=O)OCCO
L[TPA+EG]5+EG (85122-18-5)	O=C(OCCO)C1=CC=C(C=C1)C(=O)OCCOC(=O)C2=CC=C(C=C2)C(=O)OCCOC(=O)C3=CC=C(C=C3)C(=O)OCCOC(=O)C4=CC=C(C=C4)C(=O)OCCOC(=O)C5=CC=C(C=C5)C(=O)OCCO
L[TPA+EG]6+EG (85122-19-6)	O=C(C1=CC=C(C=C1)C(OCCOC(C2=CC=C(C=C2)C(OCCOC(C3=CC=C(C=C3)C(OCCOC(C4=CC=C(C=C4)C(OCCO)=O)=O)=O)=O)=O)=O)=O)OCCOC(C5=CC=C(C=C5)C(OCCOC(C6=CC=C(C=C6)C(OCCO)=O)=O)=O)=O
L[TPA+EG]+TPA (2225-05-0)	O=C(O)C1=CC=C(C=C1)C(=O)OCCOC(=O)C2=CC=C(C=C2)C(=O)O
L[TPA+EG]2+TPA (1855-25-0)	O=C(O)C1=CC=C(C=C1)C(=O)OCCOC(=O)C2=CC=C(C=C2)C(=O)OCCOC(=O)C3=CC=C(C=C3)C(=O)O
L[TPA+EG]3+TPA (122295-57-2)	O=C(O)C1=CC=C(C=C1)C(=O)OCCOC(=O)C2=CC=C(C=C2)C(=O)OCCOC(=O)C3=CC=C(C=C3)C(=O)OCCOC(=O)C4=CC=C(C=C4)C(=O)O
L[TPA+EG]4+TPA (not available)	O=C(C1=CC=C(C(OCCOC(C2=CC=C(C(OCCOC(C3=CC=C(C(OCCOC(C4=CC=C(C(OCCOC(C5=CC=C(C(O)=O)C=C5)=O)=O)C=C4)=O)=O)C=C3)=O)=O)C=C2)=O)=O)C=C1)O
L[TPA+EG]5+TPA (not available)	O=C(C1=CC=C(C(OCCOC(C2=CC=C(C(OCCOC(C3=CC=C(C(OCCOC(C4=CC=C(C(OCCOC(C5=CC=C(C(OCCOC(C6=CC=C(C(O)=O)C=C6)=O)=O)C=C5)=O)=O)C=C4)=O)=O)C=C3)=O)=O)C=C2)=O)=O)C=C1)O
L[TPA+EG]6+TPA (not available)	O=C(C1=CC=C(C=C1)C(OCCOC(C2=CC=C(C=C2)C(OCCOC(C3=CC=C(C=C3)C(OCCOC(C4=CC=C(C=C4)C(O)=O)=O)=O)=O)=O)=O)OCCOC(C5=CC=C(C=C5)C(OCCOC(C6=CC=C(C=C6)C(OCCOC(C7=CC=C(C(O)=O)C=C7)=O)=O)=O)=O)=O

Table S2. Reported concentrations ($C_{P,0}$) for PET oligomers in food contact materials. Collected information on $C_{P,0}$ values, corresponding literature source, and information if concentration was reported for the isomer as described in **Table S1** or if isomers were not defined. For values were standard deviation was available, only the mean value was captured. In case several values have been determined, the highest and lowest value were captured as a range. The highest concentration ($C_{P,0}$ max) used for migration predictions is marked in blue and the lowest concentration ($C_{P,0}$ min) is marked in green. Abbreviations: (cyclic), L (linear), TPA (terephthalic acid), EG (ethylene glycol), DEG (diethylene glycol).

PET oligomer	$C_{P,0}$ value(s) correspond(s) to isomer described in Table S1	Reported $C_{P,0}$ [mg/kg]	Literature source
C[TPA+EG]	yes	0.18 – 0.27	[2]
C[TPA+EG]2	yes	34<br>0.18 – 1.2<br>4.0×10^{-4} – 1.5×10^{-3}<br>0.40 – 25<br>30	[3]<br>[2]<br>[4]<br>[5]<br>[6]

C[TPA+EG]3	yes	2922<br>3000 – 6700<br>3000 – 4830<br>0.66 – 7.0<br>0.60 – 3.5<br>7951 – 9592<br>5150 – 8100<br>1400 – 5700<br>2.8 – 6039<br>5380 – 5680	[3]<br>[7]<br>[8]<br>[2]<br>[4]<br>[9]<br>[10]<br>[11]<br>[5]<br>[6]
C[TPA+EG]4	yes	749<br>0.61 – 0.85<br>1.0×10^{-3} – 0.4<br>450 – 1477<br>430	[3]<br>[2]<br>[4]<br>[5]<br>[6]
C[TPA+EG]5	yes	303<br>0.18 – 0.17<br>1.0×10^{-3} – 1.5×10^{-3}<br>205 – 749<br>66	[3]<br>[2]<br>[4]<br>[5]<br>[6]
C[TPA+EG]6	yes	155<br>0.10 – 1.80<br>149 – 643<br>15	[3]<br>[4]<br>[5]<br>[6]
C[TPA+EG]7	yes	1.6×10^{-3} – 2.3×10^{-3}<br>84 – 551	[12]<br>[5]
C[TPA+EG]8	yes	53 – 424	[5]
C[TPA+EG]+[TPA+DEG]	yes	281<br>2.5 – 19<br>174 – 240<br>100	[3]<br>[2]<br>[5]<br>[6]
C[TPA+EG]2+[TPA+DEG]	yes	157<br>0.34 – 2.2<br>72 – 334<br>140	[3]<br>[2]<br>[5]<br>[6]
C[TPA+EG]3+[TPA+DEG]	yes	124<br>0.18 – 0.59<br>37 – 185<br>9	[3]<br>[2]<br>[5]<br>[6]
C[TPA+EG]4+[TPA+DEG]	yes	84<br>32 – 189	[3]<br>[5]
C[TPA+EG]5+[TPA+DEG]	yes	21 – 130	[5]
C[TPA+DEG]2	yes	65<br>0.69 – 4.8	[3]<br>[2]
C[TPA+EG]+[TPA+DEG]2	yes	11 /11	[3]
C[TPA+EG]2+[TPA+DEG]2	*Isomer unclear	0.18 – 1.1*	[2]
L[TPA+EG]	yes	12.5 – 34.4<br>18 – 118	[9]<br>[5]
L[TPA+EG]2	yes	0.18 – 0.51<br>19	[2]<br>[6]
L[TPA+EG]3	yes	0.18 – 0.70<br>35	[2]<br>[6]
L[TPA+EG]4	yes	14/14	[6]
L[TPA+EG]+[TPA+DEG]	yes<br>*Isomer unclear	0.18 – 7.7<br>16 – 99*	[2]<br>[5]
L[TPA+EG]2+[TPA+DEG]	*Isomer unclear	0.18 – 1.4*<br>26 – 131*	[2]<br>[5]
L[TPA+EG]3+[TPA+DEG]	*Isomer unclear	18 – 96*	[5]
L[TPA+EG]+EG	yes	18 – 49<br>3.5 – 50	[9]<br>[5]
L[TPA+EG]2+EG	yes	38/38	[6]
L[TPA+EG]3+EG	yes	66/66	[6]
L[TPA+EG]4+EG	yes	25/25	[6]
L[TPA+EG]2+TPA	yes	7/7	[6]
L[TPA+EG]3+TPA		3/3	[6]

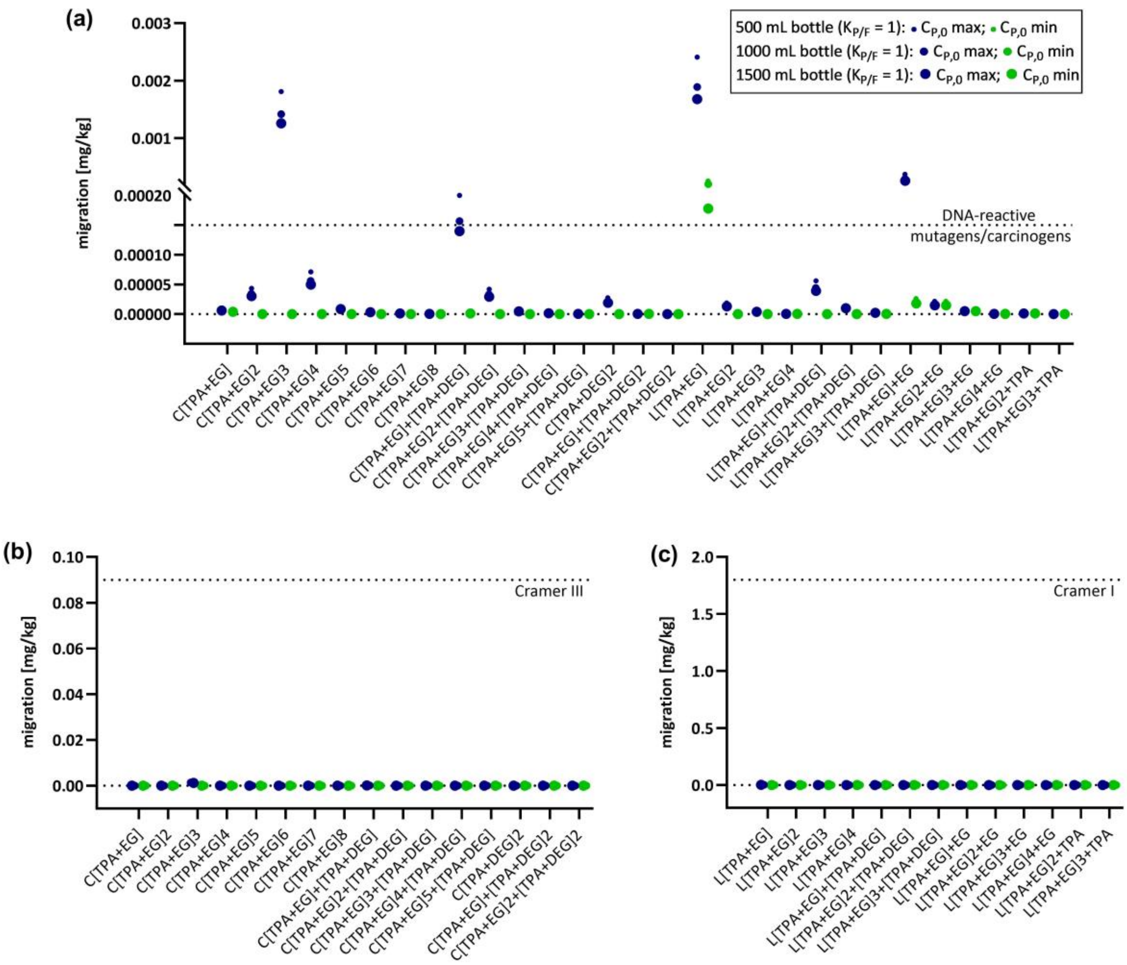

Figure S1. Graphical representation of predicted migrations using the E_A-based model. Migration of PET oligomers for which $C_{P,0}$ values were available was predicted for PET bottles (500 – 1500 mL) under long-term storage condition (25 °C, 365 d) and evaluated for the exceeding different safety thresholds. **(a)** Modeled migration of all 29 PET oligomers and their evaluation against the DNA-reactive mutagens/carcinogens safety threshold of 0.15 µg/kg. **(b)** Modeled migration of cyclic PET oligomers and their evaluation against the Cramer Class III threshold of 90 µg/kg. **(c)** Modeled migration of linear PET oligomers and their evaluation against the Cramer Class I threshold of 1.8 mg/kg.

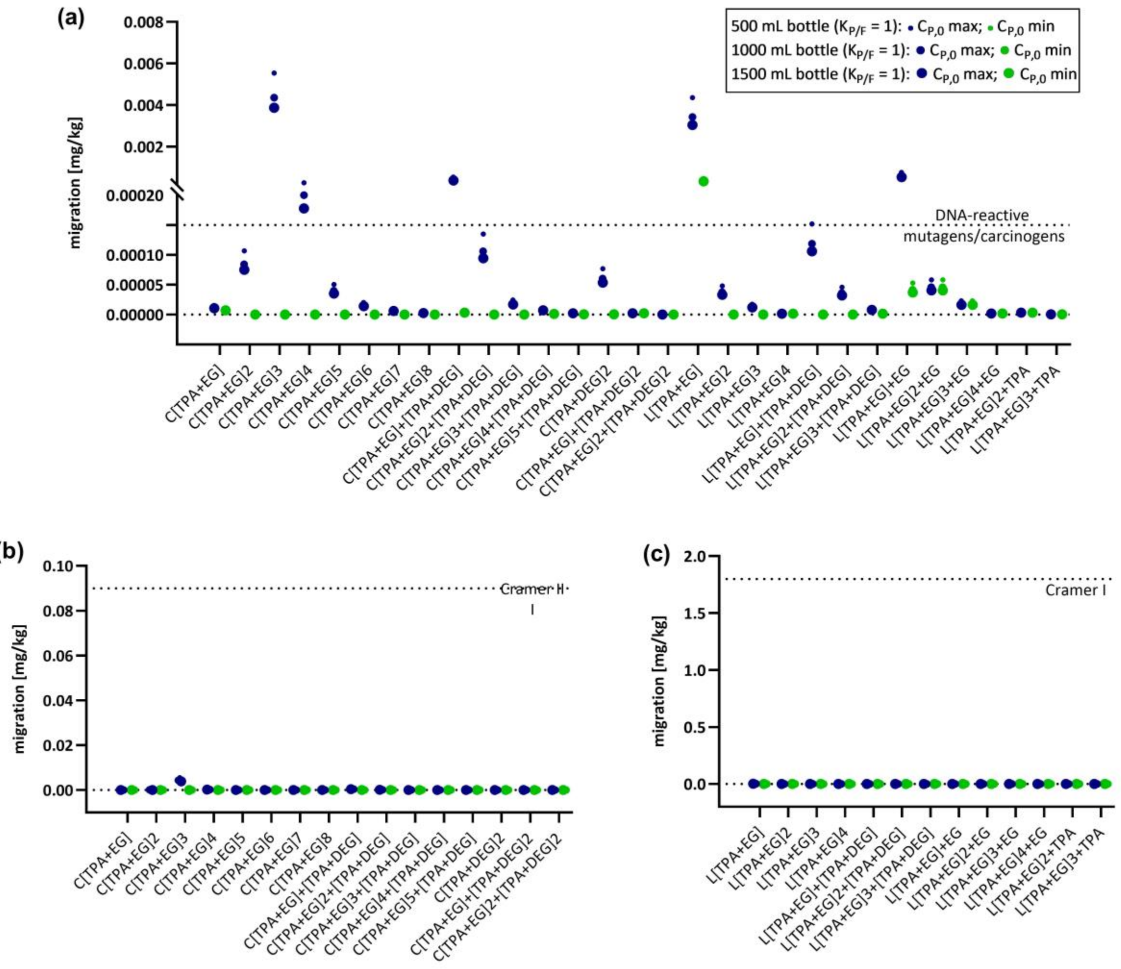

Figure S2. Graphical representation of predicted migrations using the E_A-based model. Migration of PET oligomers for which $C_{P,0}$ values were available was predicted for PET bottles (500 – 1500 mL) under short-term non-controlled/extreme summer storage condition (40 °C, 60 d) and evaluated for the exceeding different safety thresholds. **(a)** Modeled migration of all 29 PET oligomers and their evaluation against the DNA-reactive mutagens/carcinogens safety threshold of 0.15 µg/kg. **(b)** Modeled migration of cyclic PET oligomers and their evaluation against the Cramer Class III threshold of 90 µg/kg. **(c)** Modeled migration of linear PET oligomers and their evaluation against the Cramer Class I threshold of 1.8 mg/kg.

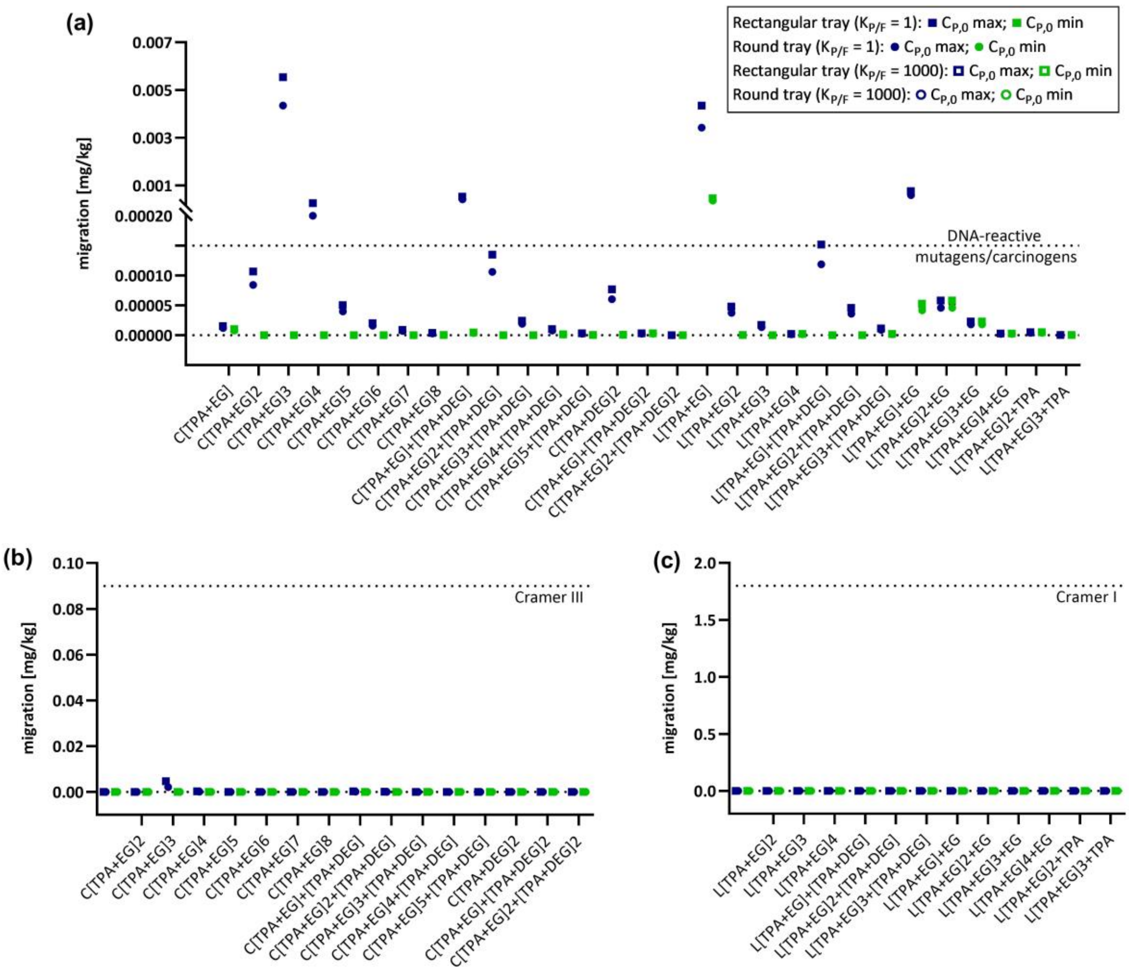

Figure S3. Graphical representation of predicted migrations using the E_A-based model. Migration of PET oligomers for which $C_{P,0}$ values were available was predicted for PET trays (rectangular and round) under short-term heating condition (70 °C, 30 min) and evaluated for the exceeding different safety thresholds. Oligomer names in bold represent molecules for which both $K_{P/F}$ = 1 and $K_{P/F}$ = 1000 were used for predictions. **(a)** Modeled migration of all 29 PET oligomers and their evaluation against the DNA-reactive mutagens/carcinogens safety threshold of 0.15 µg/kg. **(b)** Modeled migration of cyclic PET oligomers and their evaluation against the Cramer Class III threshold of 90 µg/kg. **(c)** Modeled migration of linear PET oligomers and their evaluation against the Cramer Class I threshold of 1.8 mg/kg.

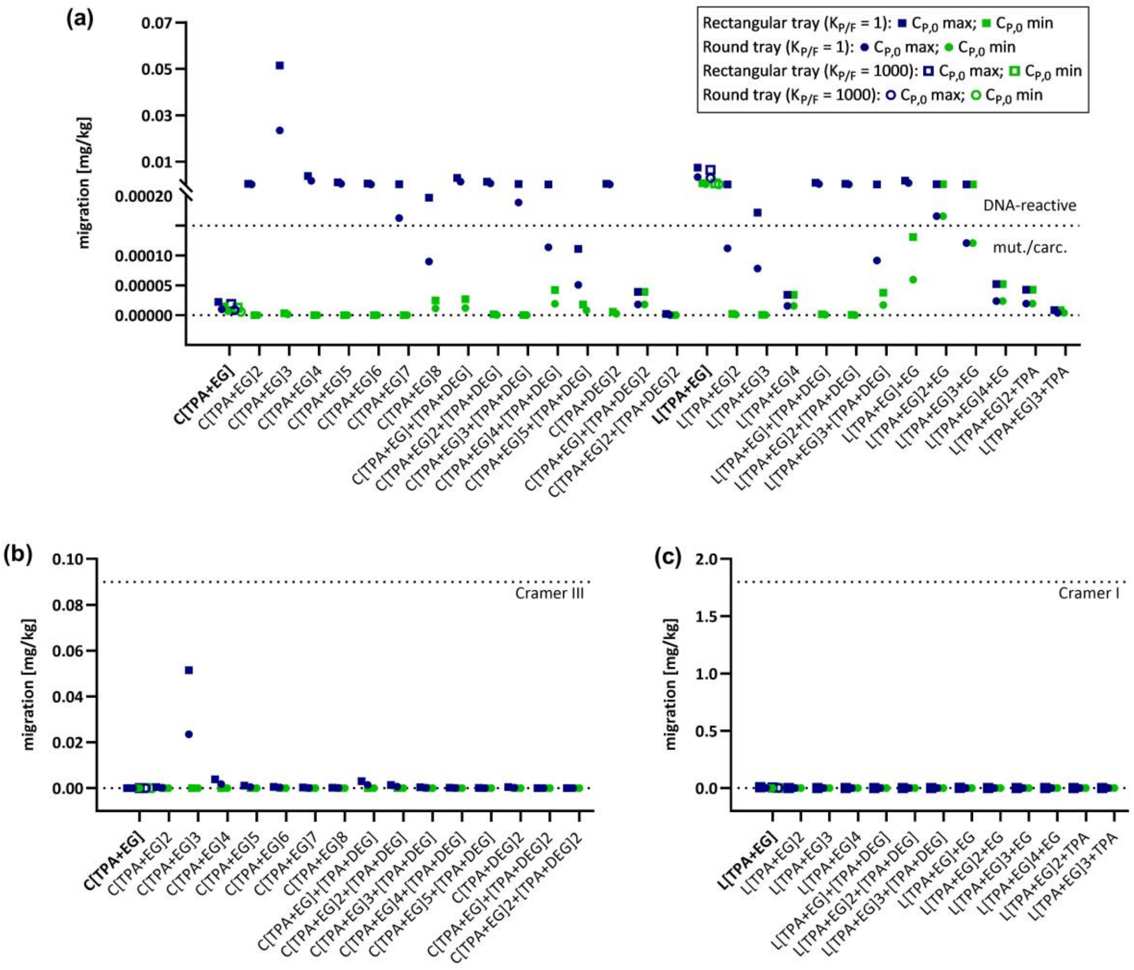

Figure S4. Graphical representation of predicted migrations using the E_A-based model. Migration of PET oligomers for which $C_{P,0}$ values were available was predicted for PET trays (rectangular and round) under short-term heating condition (100 °C, 10 min) and evaluated for the exceeding different safety thresholds. Oligomer names in bold represent molecules for which both $K_{P/F}$ = 1 and $K_{P/F}$ = 1000 were used for predictions. **(a)** Modeled migration of all 29 PET oligomers and their evaluation against the DNA-reactive mutagens/carcinogens safety threshold of 0.15 µg/kg. **(b)** Modeled migration of cyclic PET oligomers and their evaluation against the Cramer Class III threshold of 90 µg/kg. **(c)** Modeled migration of linear PET oligomers and their evaluation against the Cramer Class I threshold of 1.8 mg/kg.

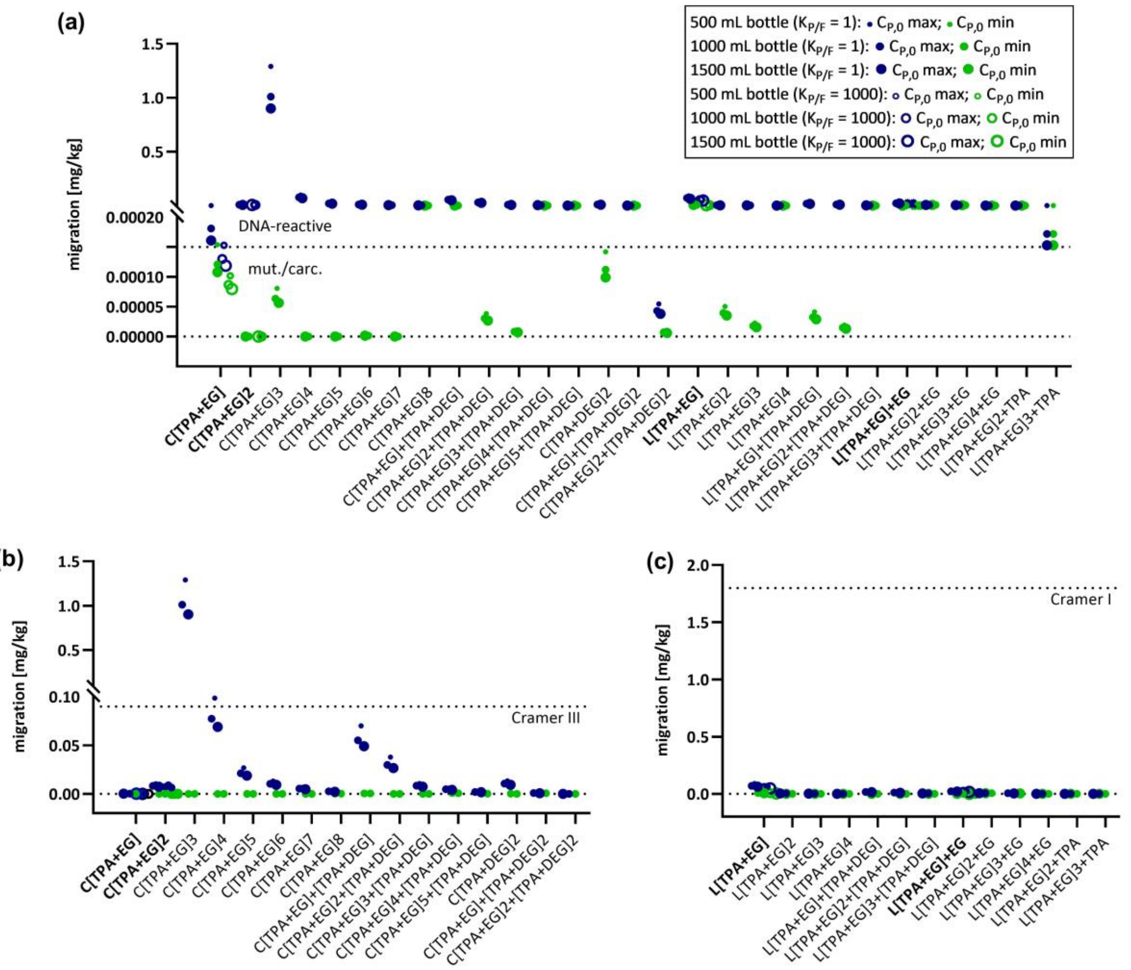

Figure S5. Graphical representation of predicted migrations using the A_P model. Migration of PET oligomers for which $C_{P,0}$ values were available was predicted for PET bottles (500 – 1500 mL) under long-term storage condition (25 °C, 365 d) and evaluated for the exceeding different safety thresholds. Oligomer names in bold represent molecules for which both $K_{P/F}$ = 1 and $K_{P/F}$ = 1000 were used for predictions. **(a)** Modeled migration of all 29 PET oligomers and their evaluation against the DNA-reactive mutagens/carcinogens safety threshold of 0.15 µg/kg. **(b)** Modeled migration of cyclic PET oligomers and their evaluation against the Cramer Class III threshold of 90 µg/kg. **(c)** Modeled migration of linear PET oligomers and their evaluation against the Cramer Class I threshold of 1.8 mg/kg.

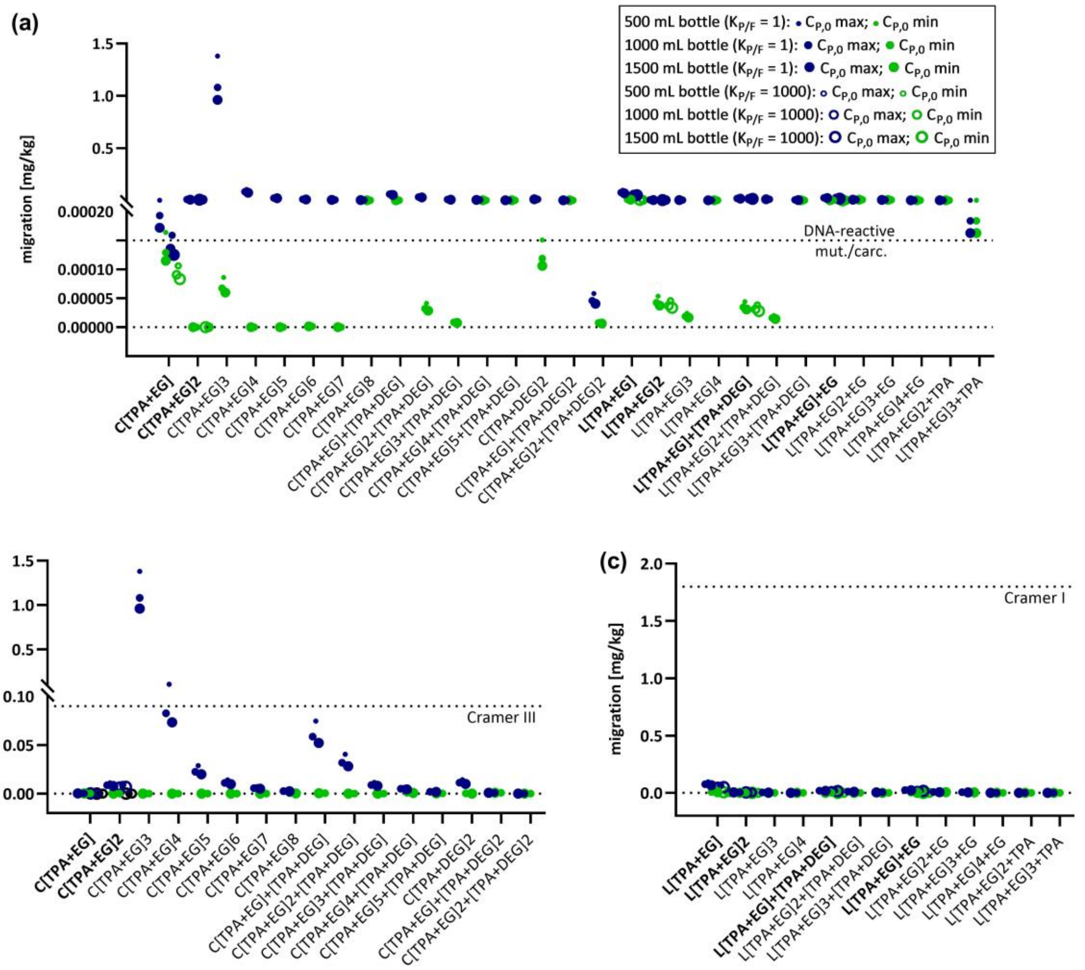

Figure S6. Graphical representation of predicted migrations using the A_P model. Migration of PET oligomers for which $C_{P,0}$ values were available was predicted for PET bottles (500 – 1500 mL) under short-term non-controlled/extreme summer storage condition (40 °C, 60 d) and evaluated for the exceeding different safety thresholds. Oligomer names in bold represent molecules for which both $K_{P/F} = 1$ and $K_{P/F} = 1000$ were used for predictions. **(a)** Modeled migration of all 29 PET oligomers and their evaluation against the DNA-reactive mutagens/carcinogens safety threshold of 0.15 µg/kg. **(b)** Modeled migration of cyclic PET oligomers and their evaluation against the Cramer Class III threshold of 90 µg/kg. **(c)** Modeled migration of linear PET oligomers and their evaluation against the Cramer Class I threshold of 1.8 mg/kg.

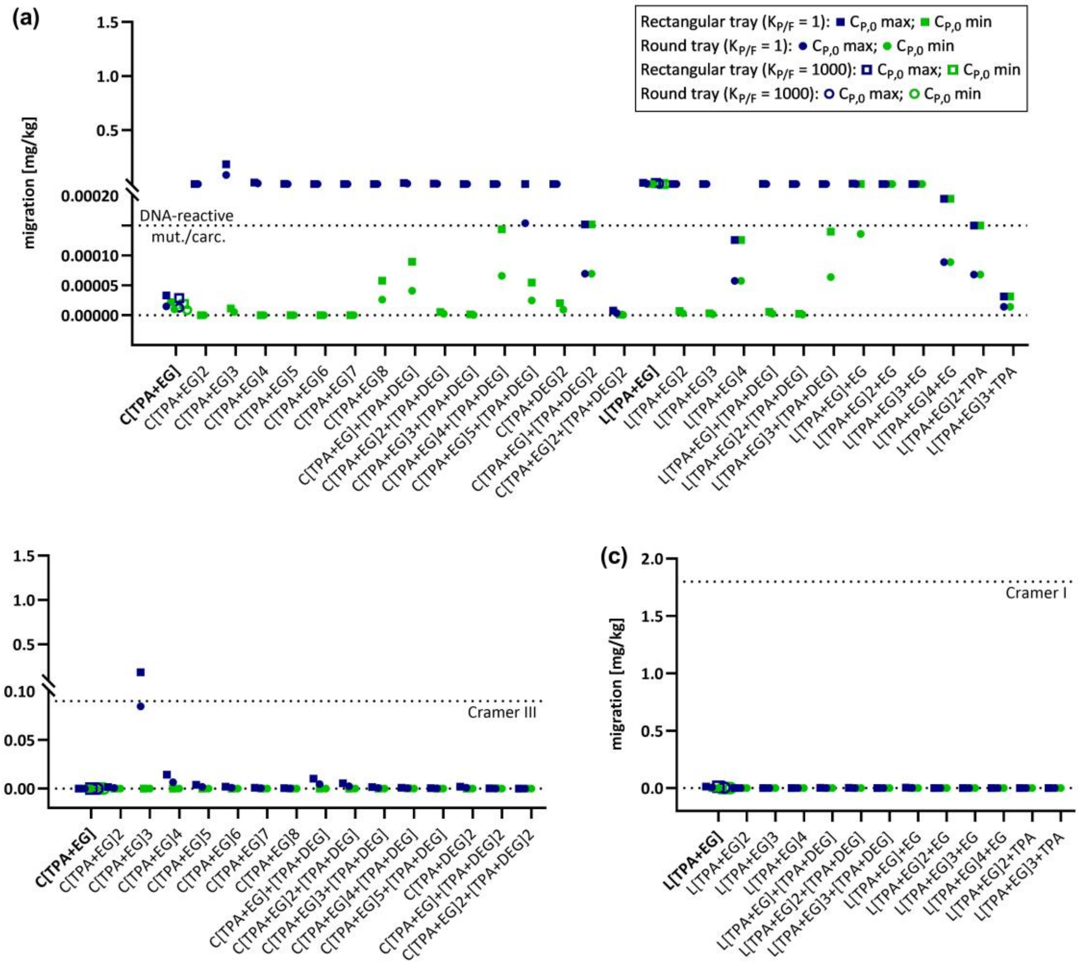

Figure S7. Graphical representation of predicted migrations using the A_P model. Migration of PET oligomers for which $C_{P,0}$ values were available was predicted for PET trays (rectangular and round) under short-term heating condition (70 °C, 30 min) and evaluated for the exceeding different safety thresholds. Oligomer names in bold represent molecules for which both $K_{P/F}$ = 1 and $K_{P/F}$ = 1000 were used for predictions. **(a)** Modeled migration of all 29 PET oligomers and their evaluation against the DNA-reactive mutagens/carcinogens safety threshold of 0.15 µg/kg. **(b)** Modeled migration of cyclic PET oligomers and their evaluation against the Cramer Class III threshold of 90 µg/kg. **(c)** Modeled migration of linear PET oligomers and their evaluation against the Cramer Class I threshold of 1.8 mg/kg.

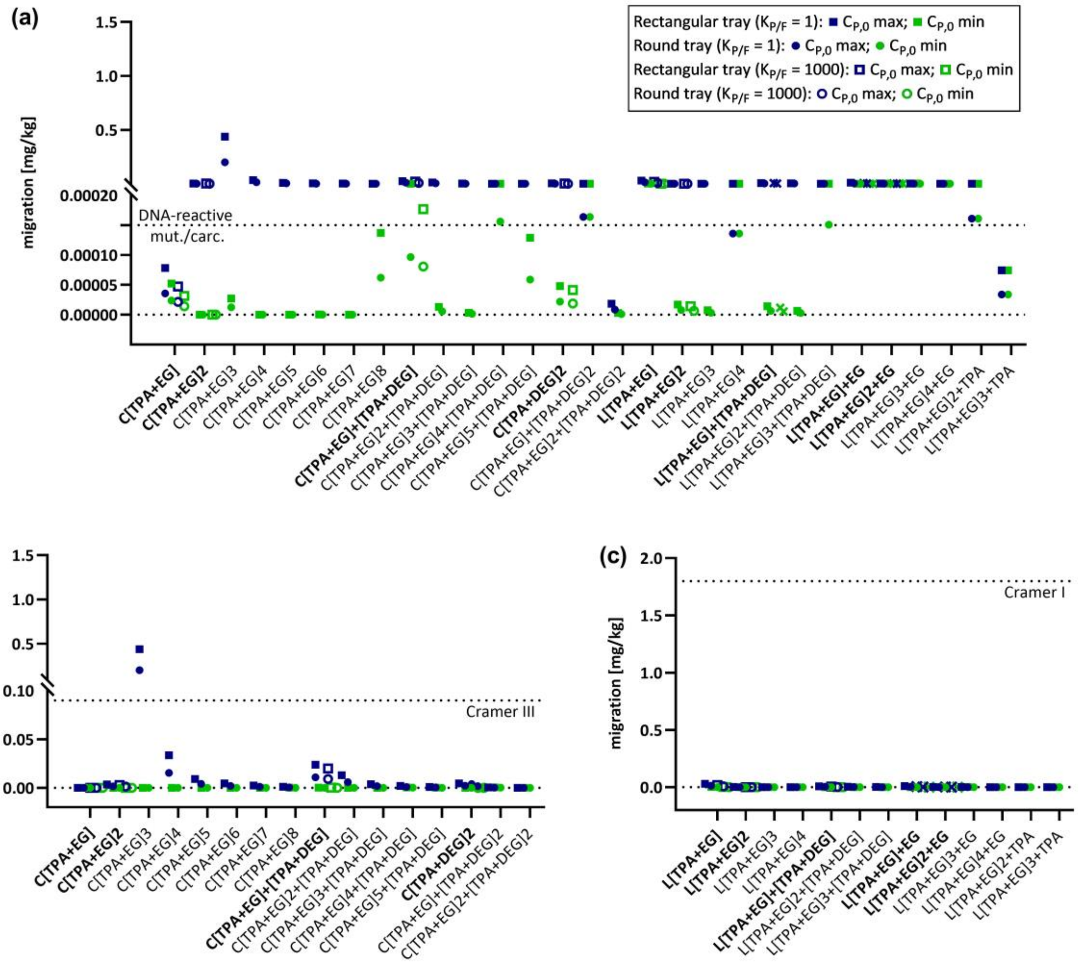

Figure S8. Graphical representation of predicted migrations using the A_P model. Migration of PET oligomers for which $C_{P,0}$ values were available was predicted for PET trays (rectangular and round) under short-term heating condition (100 °C, 10 min) and evaluated for the exceeding different safety thresholds. Oligomer names in bold represent molecules for which both $K_{P/F}$ = 1 and $K_{P/F}$ = 1000 were used for predictions. **(a)** Modeled migration of all 29 PET oligomers and their evaluation against the DNA-reactive mutagens/carcinogens safety threshold of 0.15 µg/kg. **(b)** Modeled migration of cyclic PET oligomers and their evaluation against the Cramer Class III threshold of 90 µg/kg. **(c)** Modeled migration of linear PET oligomers and their evaluation against the Cramer Class I threshold of 1.8 mg/kg.

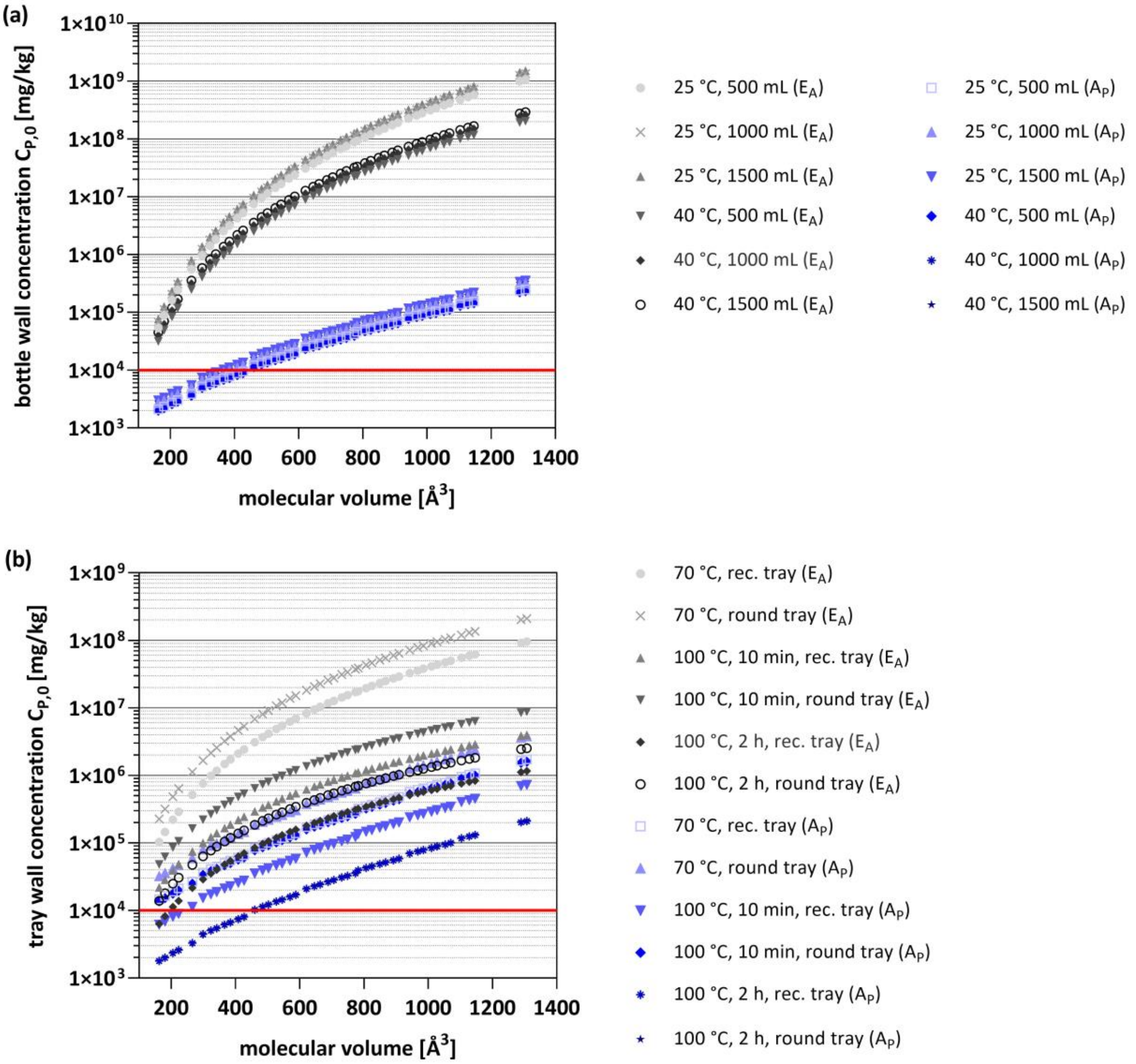

Figure S9. Maximum bottle and tray wall concentrations ($C_{P,0}$) of all oligomers corresponding to a migration of 1.8 mg/kg, calculated for the E_A-based model (grey, (E_A)) and the A_P model (blue, (A_P)). **(a)** $C_{P,0}$ concentrations for 500, 1000, and 1500 mL bottle at 25 °C for 365 d and 40 °C for 60 d. **(b)** $C_{P,0}$ concentrations for rectangular (rec.) and round trays at 70 °C for 30 min and 100 °C for 10 min or 2 h. Red line indicates a $C_{P,0}$ concentration of 1% (w/w).

References

1. Schreier, V.N.; Appenzeller-Herzog, C.; Brüschweiler, B.J.; Geueke, B.; Wilks, M.F.; Simat, T.J.; Schilter, B.; Smieško, M.; Muncke, J.; Odermatt, A.; et al. Evaluating the Food Safety and Risk Assessment Evidence-Base of Polyethylene Terephthalate Oligomers: Protocol for a Systematic Evidence Map. *Environ Int* **2022**, *167*, doi:10.1016/J.ENVINT.2022.107387.
2. Ubeda, S.; Aznar, M.; Nerín, C. Determination of Oligomers in Virgin and Recycled Polyethylene Terephthalate (PET) Samples by UPLC-MS-QTOF. *Anal Bioanal Chem* **2018**, *410*, 2377–2384, doi:10.1007/s00216-018-0902-4.
3. Hoppe, M.; Fornari, R.; de Voogt, P.; Franz, R. Migration of Oligomers from PET: Determination of Diffusion Coefficients and Comparison of Experimental versus Modelled Migration. *Food Additives & Contaminants: Part A* **2017**, *34*, 1251–1260, doi:10.1080/19440049.2017.1322222.
4. Alberto Lopes, J.; Tsochatzis, E.D.; Karasek, L.; Hoekstra, E.J.; Emons, H. Analysis of PBT and PET Cyclic Oligomers in Extracts of Coffee Capsules and Food Simulants by a HPLC-UV/FLD Method. *Food Chem* **2021**, *345*, 128739, doi:10.1016/j.foodchem.2020.128739.
5. Kim, D.J.; Lee, K.T. Determination of Monomers and Oligomers in Polyethylene Terephthalate Trays and Bottles for Food Use by Using High Performance Liquid Chromatography-Electrospray Ionization-Mass Spectrometry. *Polym Test* **2012**, *31*, 490–499, doi:10.1016/J.POLYMERTESTING.2012.02.001.
6. Dulio, V.; Po, R.; Borrelli, R.; Guarini, A.; Santini, C. Characterization of Low-Molecular-Weight Oligomers in Recycled Poly(Ethylene Terephthalate). *Die Angewandte Makromolekulare Chemie* **1995**, *225*, 109–122, doi:10.1002/APMC.1995.052250110.
7. Mutsuga, M.; Tojima, T.; Kawamura, Y.; Tanamoto, K. Survey of Formaldehyde, Acetaldehyde and Oligomers in Polyethylene Terephthalate Food-Packaging Materials. *Food Addit Contam* **2007**, *22*, 783–789, doi:10.1080/02652030500157593.
8. Nasser, A.L.M.; Lopes, L.M.X.; Eberlin, M.N.; Monteiro, M. Identification of Oligomers in Polyethyleneterephthalate Bottles for Mineral Water and Fruit Juice: Development and Validation of a High-Performance Liquid Chromatographic Method for the Determination of First Series Cyclic Trimer. *J Chromatogr A* **2005**, *1097*, 130–137, doi:10.1016/j.chroma.2005.08.023.
9. Begley, T.H.; Hollifield, H.C. Liquid Chromatographic Determination of Residual Reactants and Reaction By-Products in Polyethylene Terephthalate. *J AOAC Int* **1989**, *72*, 468–470, doi:10.1093/JAOAC/72.3.468.
10. Begley, T.H.; Hollifield, H.C. Evaluation of Polyethylene Terephthalate Cyclic Trimer Migration from Microwave Food Packaging Using Temperature-time Profiles. *Food Addit Contam* **2009**, *7*, 339–346, doi:10.1080/02652039009373898.
11. Ohkado, Y.; Kawamura, Y.; Mutsuga, M.; Tamura, H.O.; Tanamoto, K. Analysis of Formaldehyde, Acetaldehyde and Oligomers in Recycled Polyethylene Terephthalate. *Shokuhin Eiseigaku Zasshi* **2005**, *46*, 218–223, doi:10.3358/SHOKUEISHI.46.218.
12. Begley, T.H.; Hollifield, H.C. High-Performance Liquid Chromatographic Determination of Migrating Poly(Ethylene Terephthalate) Oligomers in Corn Oil. *J Agric Food Chem* **2002**, *38*, 145–148, doi:10.1021/JF00091A031.
